# Preparation and evaluation of crocin loaded in nanoniosomes and their effects on ischemia–reperfusion injuries in rat kidney

**DOI:** 10.1038/s41598-021-02073-w

**Published:** 2021-12-07

**Authors:** Reyhaneh Naderi, Abbas Pardakhty, Mohammad Farajli Abbasi, Mehdi Ranjbar, Maryam Iranpour

**Affiliations:** 1 Pharmaceutics Research Center, Institute of Neuropharmacology, Kerman University of Medical Sciences, P.O. Box: 76175-493, 76169-11319 Kerman, Iran; 2 Neuroscience Research Center, Institute of Neuropharmacology, Kerman University of Medical Sciences, Kerman, Iran; 3 Department of Pathology, Pathology and Stem Cell Research Center, Afzalipour Faculty of Medicine, Kerman University of Medical Sciences, Kerman, Iran

**Keywords:** Biochemistry, Biological techniques, Drug discovery, Neuroscience

## Abstract

As a powerful antioxidant compound, crocin can partially protect against renal ischemia/reperfusion (I/R) injuries. The encapsulation of components in niosomes (non-ionic surfactant-based vesicle) as nano-sized carrier systems has been proposed as they improve the solubility, stability, and bioavailability of drugs. Herein, the encapsulation of crocin in nano-niosomes and the effects of crocin-loaded nano-niosomes on renal ischemia/reperfusion-induced damages were evaluated. Nano-niosomes containing crocin were formulated by a modified heating method and were characterized for their physicochemical characteristics. Ischemia was induced by clamping the renal artery for 30 min followed by 1 or 24 h of reperfusion. Rats received an intra-arterial injection of nano-niosome-loaded crocin at the outset of reperfusion. Blood samples were taken after reperfusion to measure urea, creatinine (Cr), malondialdehyde (MDA), and superoxide dismutase (SOD) activity. The right kidney was removed for histological examination. The results showed that crocin-contain nano-niosomes have appropriate size and morphology, acceptable encapsulation efficiency, and a proper release pattern of crocin. I/R enhanced creatinine (Cr), urea, and malondialdehyde (MDA) serum levels and reduced SOD activity and histological damages in the renal tissue.

## Introduction

Renal ischemia–reperfusion (RIR) is a major clinical problem caused by temporary ligation of renal blood vessels. Restriction of blood flow to the kidneys occurs in various clinical situations, including partial nephrectomy, sepsis, hypotension, and kidney transplantation^1,2^. Numerous studies have demonstrated that renal I/R injury is the main cause of acute kidney injury (AKI)^3^. The reperfusion of blood flow to the ischemic kidney is shown to increase the production of oxygen free radicals, thereby leading to further ischemic renal injury^4^. Furthermore, a defect in the antioxidant defense system could be involved in the pathogenesis of renal ischemia–reperfusion injury.

Nanotechnology-based delivery carriers such as nanoparticles, liposomes, niosomes, micelles, and solid lipid particles have attracted attention as carriers in delivering therapeutic agents. There are various advantages to nano-carriers, e.g., prolonging the half-life of drugs, reducing non-specific adsorption, improving the serum solubility of drugs, and preferentially delivering drugs to the tissues^5,6^. Niosomes are vesicular nano-carriers composed of non-ionic surfactants utilized as novel drug delivery systems. The core–shell structure of niosomes allows the encapsulation of both hydrophilic and lipophilic substances. Niosomes are also recognized for their high chemical stability, low cost, biodegradability, low toxicity, and easy handling and storage^7,8^.

Saffron (the dried stigmata of the flowers of *Crocus sativus*) has been consumed as a spice as well as in folk medicine since ancient times. Its protective effects result from its active components, e.g., crocin and crocetin. Crocin, a water-soluble carotenoid, is the most important and abundant antioxidant compound of saffron^9^. Previous studies have demonstrated that crocin exerts different pharmacological effects such as anti-oxidant^10,11^, anti-inflammatory^12^, cancer cell proliferation inhibiting^13^, hypolipidemic^14^, antianxiety^15^, antidepressant^16^, and memory and learning enhancing effects^17^. Crocin is reported to improve ischemia/reperfusion-induced injuries in various tissues, including the brain^18^, skeletal muscles^19^, heart^20^, retina^21^, and kidneys^22^. Its protective effects on renal failure through its antioxidant characteristics are also documented. The oral administration of crocin enhances SOD and total antioxidant capacity of the kidneys and reduces the level of MDA serum in mice^23^. It also decreased blood urea, creatinine, urinary glucose, and protein concentrations. In addition, pretreatment with crocin increased total thiol and glutathione peroxidase concentrations in rats^24^. There are reports that crocin acts as an antioxidant in kidneys and can improve renal I/R injuries^22–24^. In addition, niosomes have recently been used as new lipid-based nano-carriers for the delivery of various drugs. The present study was conducted to formulate crocin-containing nano-niosomes and evaluate their possible protective effects on ischemia–reperfusion-induced damage on kidneys.

## Experimental procedure

### Materials and methods

The mean size and size distribution of nano-niosomes were measured using a dynamic light scattering technique via a Malvern Nano ZS light scattering apparatus (Worcestershire, Malvern Instruments Ltd., UK). Scanning electron microscopy (SEM) was used to determine the morphology of crocin-loaded nano-niosomes. Transmission electron microscopy (TEM) images were evaluated with a Philips EM_2_08S equipped with an accelerating voltage of 100 kV. Different concentrations of crocin in the PBS were prepared. Then, the absorption rate of each sample was measured using a UV–visible spectrophotometer. The standard curve was plotted by using different concentrations of crocin and the obtained absorption wavelengths, λmax = 440 nm. A Shimadzu Varian 4300 spectrophotometer in KBr pellets was employed for measuring the Fourier-transform infrared (FT-IR) spectra. All the methods were performed in accordance with the relevant guidelines and regulations. This study is reported following the ARRIVE guidelines.

### Preparation of crocin-loaded nano-niosomes

Nano-niosomes were formulated using the heating method with some modifications^25^. To this end, cholesterol and Tween 40 were first dissolved in glycerol and heated at elevated temperatures (120 °C) while stirring (approx. 1000 rpm) for 15 min. Crocin dissolved in the aqueous phase (normal saline) was then added to a preheated mixture of cholesterol, Tween, and glycerol. The final mixture was stirred on a hotplate at 1000 rpm and 80 °C for 30 min. All the reactions were performed in an eight-baffled glass vessel. The niosomal samples were kept at room temperature for 24 h after preparation for stabilization.

### Animals and experimental groups

Adult male Wistar rats weighing 180–200 g were caged in a photo period controlled room (12-h light/dark cycle) at 22 ± 2 °C. Food and water were available ad libitum. All the experimental procedures were approved by the Animal Research Ethics Committee of the Kerman Neuroscience Research Center. The rats were randomly divided into the following groups: the control group not subjected to the ischemia–reperfusion; the I/R group subjected to 30 min of ischemia and 1 h or 24 h of reperfusion; empty niosome-treated group subjected to 30 min of ischemia followed by empty niosome intravenous administration and 1 h or 24 h of reperfusion; crocin-treated group subjected to 30 min of ischemia followed by crocin-contain nano-niosome intravenous administration and 1 h or 24 h of reperfusion. The male rats were obtained from the Kerman University of Medical Sciences animal farm. This study received ethical approval from the local ethical committee of the Kerman University of Medical Sciences as a thesis at the Faculty of Pharmacy.

### Encapsulation efficiency determination

The capacity of nano-niosomes to entrap crocin was determined by separating the un-entrapped crocin from the encapsulated one using a centrifuge (WiseSpin, Korea) at 13,000 rpm for 15 min. The clear supernatant was removed, and the pellet was washed with PBS to remove non-entrapped drugs. The concentration of the free drug in the nano-niosomes was determined by spectrophotometry at 440 nm. The encapsulation efficiency was calculated using the following equation:

1 $$ EE\% =Drug \, in \, the \, nano{\text{-}}niosomes/Total \, drug \times \, 100 $$

The release pattern of crocin from nano-niosomes was investigated using the membrane dialysis technique. The membrane was fixed between donor and receptor compartments of the static vertical diffusion Franz cell, which was continuously shaken at 500 rpm and 37 °C. One mL of the nano-noisome suspension was poured into the donor compartment and the receptor compartment was filled with 15 mL of normal saline. At specific time intervals, 1 mL of the sample was withdrawn from the receptor compartment and replaced with an equal volume of fresh normal saline. The concentration of crocin released from the nano-niosomes was measured using a spectrophotometer at 440 nm. The percentage of released crocin was estimated by the standard curve.

### Kidney ischemia

The animals were anesthetized via intraperitoneal injection of xylazine (10 mg/kg) and ketamine (70 mg/kg). The abdominal region was cleaned and sterilized with povidone-iodine solution. A midline abdominal incision was made, and the right renal pedicle was exposed. The renal artery was clamped for 30 min with atraumatic vascular clamps to induce ischemia, followed by 1 or 24 h of reperfusion. The crocin nano-niosomes were administered at the outset of reperfusion through the renal artery. After reperfusion, the blood samples were collected and frozen for biochemical analysis. The right kidney was also removed and stored in 10% formalin for histological examination. The measurement of renal functional parameters, including urea and creatinine (Cr) in the serum samples was performed by colorimetric methods using commercial kits according to their manufacturers' instructions.

### Determination of antioxidant enzymes

Malondialdehyde levels were evaluated in the kidney homogenates by the thiobarbituric acid (TBA) method. MDA reacts with thiobarbituric acid to produce a pink color with maximum absorption at 532 nm. The superoxide dismutase activity of the kidney tissue was determined by a commercial kit according to the manufacture's instruction. The SOD activity is expressed as U per mg protein.

### Histopathological examinations

The kidney tissues fixed in formalin were dehydrated in graded concentrations of alcohols and embedded in paraffin. Tissue blocks were cut into 5 μm sections using a microtome and stained with hematoxylin and eosin (H&E). The tissue sections were evaluated for the size of the glomerular tuft, necrosis, and exfoliation of the proximal tubular cells into the lumen, dilatation and congestion of blood vessels, medullary vascular congestion, inflammation, and tubular cast under a light microscope.

### Statistical analysis

The data were analyzed using SPSS 16 and expressed as means ± SEM. Data related to urea, Cr, MDA, and SOD were analyzed using one-way analysis of variance (ANOVA) followed by Tukey’s post-hoc test. Repeated measures ANOVA was used to compare the data of ischemia followed by 1 h and 24 h of reperfusion. P < 0.05 was set as the criterion for statistical significance. The histopathological data were compared between groups by Kruskal–Wallis multiple comparison test, and the Mann–Whitney U test was run for binary comparisons.

## Results and discussion

### Characterization

**Figure 1:**
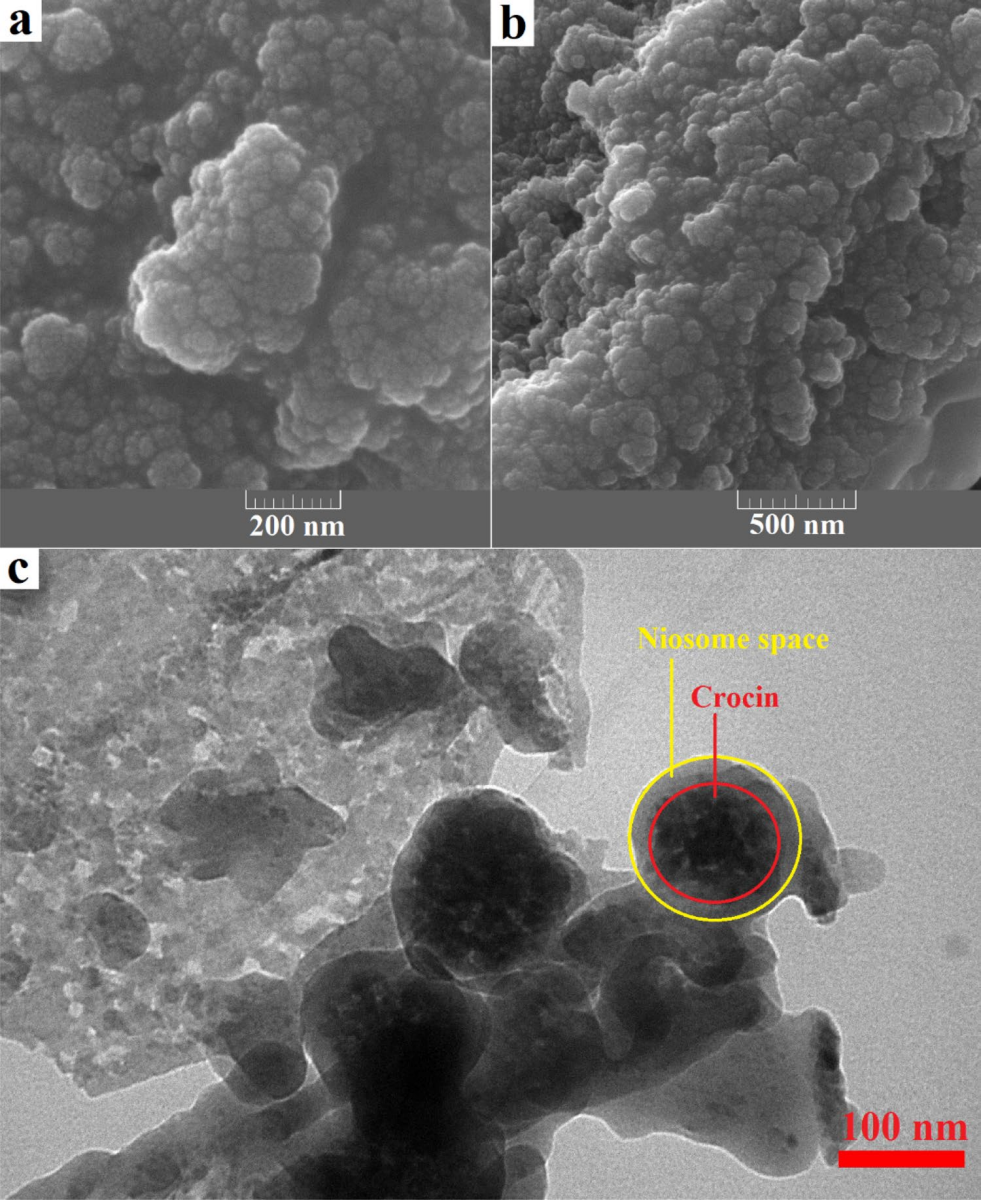
SEM images of the niosomal formulation contain crocin at scale 200 nm (**a**), scale 500 nm (**b**) and TEM image (**c**).

The surface properties of nano-niosomes were evaluated with scanning electron microscopy images through a focused beam of electrons on the sample surface. Crocin structures were trapped in nano-niosomes as scattered and concentrated in some areas. The size of the niosomal formulation containing crocin was approximately 80–150 nm. Figure 1a,b depict the SEM images of the niosomal formulation containing crocin at two scales of 200 nm and 500 nm, respectively. Transmission electron microscopy (TEM) images can be used to characterize nano-niosomes after loading. As shown in the TEM image, the dense areas can be related to crocin drugs trapped in nano-niosome structures. Figure 1c illustrates the TEM image of crocin structures in the niosomal formulation. TEM analysis confirmed the approximate shape of the final products with a diameter of < 150 nm.

**Figure 2:**
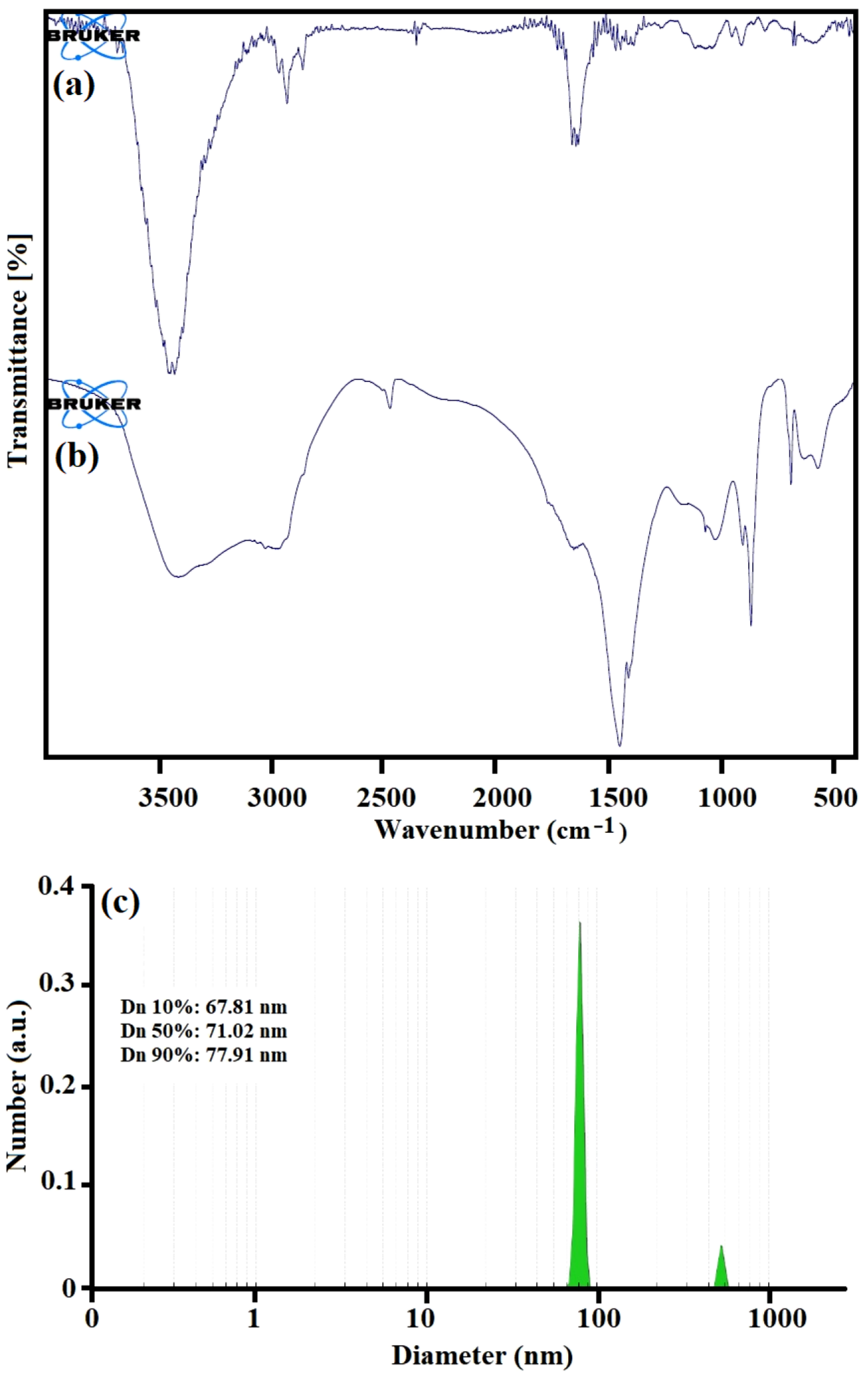
The FT-IR spectroscopy of the niosomal formulation (**a**), the niosomal formulation contain crocin (**b**) and DLS analysis.

Fourier-transform infrared spectroscopy (FTIR) was employed to investigate the formation of the final products and the characterization of functional groups. The strong peaks related to C–H stretching (2800–3000 cm^−1^) and C=O stretching (1731 cm^−1^) at niosomes can be seen in Fig. 2a. The peaks in the area of 1171 cm^−1^ and under 1000 cm^−1^ can be attributed to (–C–CO–O–) and aliphatic C–H stretching due to the presence of surfactants. The band appearing at 1701 cm^−1^ can be assigned to the carbonyl group (C=O) and the C–O stretching vibration at 1229 cm^−1^. The strong absorption peak at 1079 cm^−1^ is in accordance with to C–O bands, which can be attributed to the sugar functional groups in crocin structures [1111]. The FT-IR spectroscopy of the final product is displayed in Fig. 2b. DLS analysis as a non-destructive technique was used to investigate the particle size. The mean size of crocin loaded in nano-niosomes was about 60–80 nm. Distribution statistics such as distribution number (Dn) for the crocin loaded in nano-niosomes were calculated as Dn 10%: 67.81 nm, Dn 50%: 71.02 nm, and Dn 90%: 77.91 nm. Figure 2c illustrates the DLS analysis for the niosomal formulation containing crocin.

### Encapsulation efficiency

**Figure 3:**
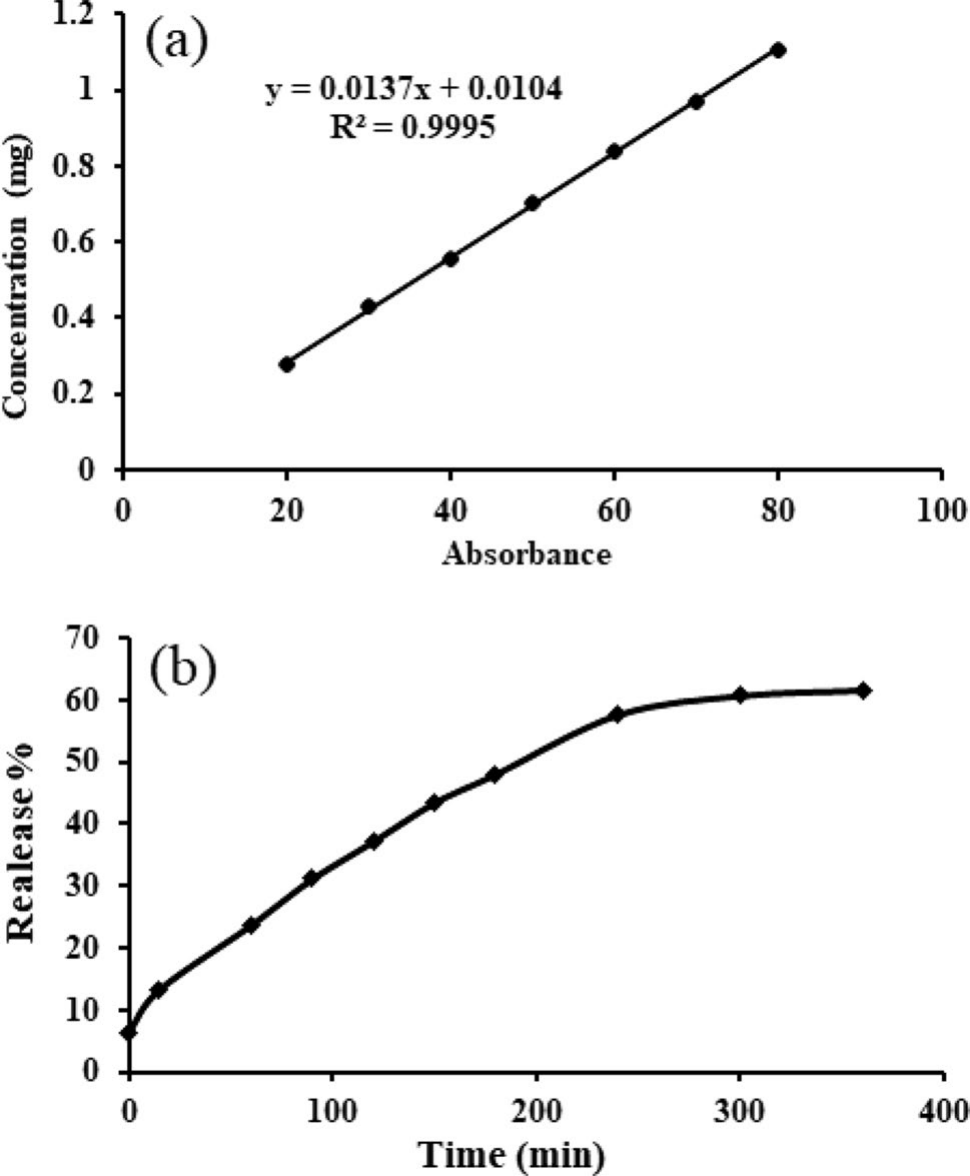
Standard calibration curve of the crocin (**a**) and the pattern of drug release of the niosomal formulation contain crocin.

The total content of free and nano-niosomal crocin was analyzed and is expressed in terms of crocin equivalent (the standard curve equation: Y = 0.0137x + 0.0104, R^2^ = 0.9995, mean ± SD, n = 3). The encapsulation percentage was calculated according to the standard curve. The results showed that the encapsulation efficiency of crocin-containing nano-niosomes was 45.8%. The release rate of crocin from the nano-niosomes was calculated by dialysis bag over different time intervals using the standard crocin curve. Figure 3a,b show the standard calibration curve of crocin and the pattern of drug release of the crocin-containing niosomal formulation, respectively. According to the diagram, the maximum amount of drug released from the niosomes within 6 h was about 61.55%. Furthermore, the release of crocin from the nano-particles was controlled and slow.

### The effect of crocin-loaded nano-niosomes on serum creatinine and urea levels

**Figure 4:**
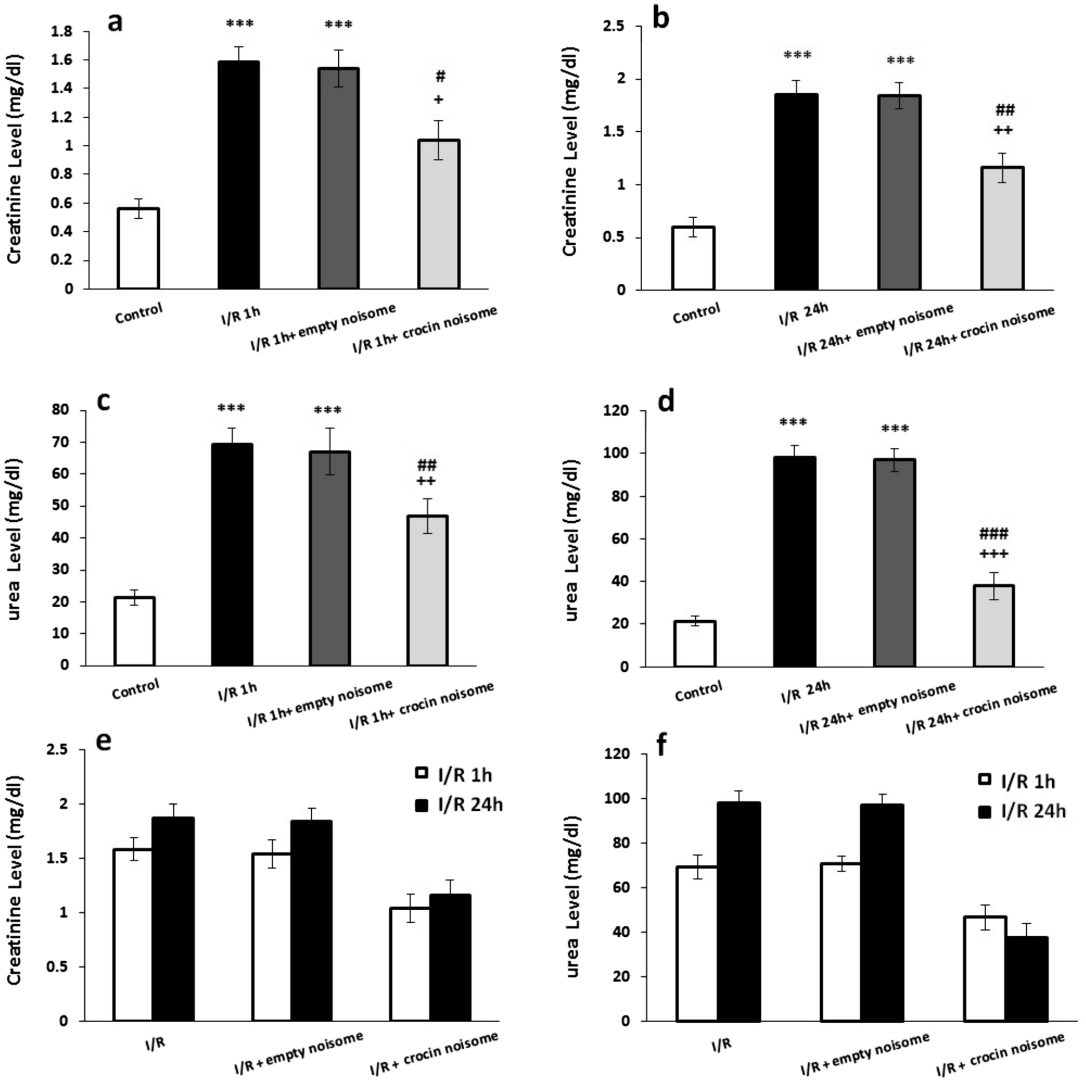
The effect of crocin-loaded nanoniosomes on (**a**) 1 h after I/R-induced changes in creatinine serum level, (**b**) 24 h after I/R-induced changes in creatinine serum level, (**c**) 1 h after I/R-induced changes in urea serum level, (**d**) 24 h after I/R-induced changes in urea serum level, (**e**) comparison ischemia-induced changes followed by 1 h and 24 of reperfusion in creatinine serum level and (**f**) comparison ischemia-induced changes followed by 1 h and 24 of reperfusion in urea serum level. Data presented as mean ± SEM. ***P < 0.001 and versus control group; ^+^P < 0.05, ^++^P < 0.01and ^+++^P < 0.001 versus I/R group; ^#^P < 0.05, ^##^P < 0.01 and ^###^P < 0.001 versus I/R + empty niosome group.

Figure 4 displays that 30 min of renal ischemia followed by 1 h of reperfusion significantly increased serum creatinine and urea levels in I/R compared to the control group (P < 0.001). Crocin-loaded nano-niosomes significantly attenuated the I/R-induced rise of creatinine (P < 0.05, Fig. 4a) and urea (P < 0.01, Fig. 4c) levels in the rat serum. In addition, ischemia followed by 24 h of reperfusion caused a significant elevation in the serum concentration of creatinine and urea compared to the control animals (P < 0.001). The concentrations of serum creatinine (P < 0.01, Fig. 4b) and urea (P < 0.001, Fig. 4d) were significantly decreased in rats treated with crocin-contain nano-niosomes compared with the untreated groups. There was no significant difference between I/R group (P = 0.468), I/R + empty niosome (P = 0.771) and I/R + crocin noisome (P = 0.971) groups in creatinine level after ischemia followed by 1 h and 24 h of reperfusion (Fig. 4e). In addition, no significant difference was observed between I/R group (P = 0.578), I/R + empty niosome (P = 0.235) and I/R + crocin noisome (P = 0.541) groups in urea level after ischemia followed by 1 h and 24 h of reperfusion (Fig. 4f).

### The effect of crocin-loaded nano-niosomes on serum SOD and MDA levels

**Figure 5:**
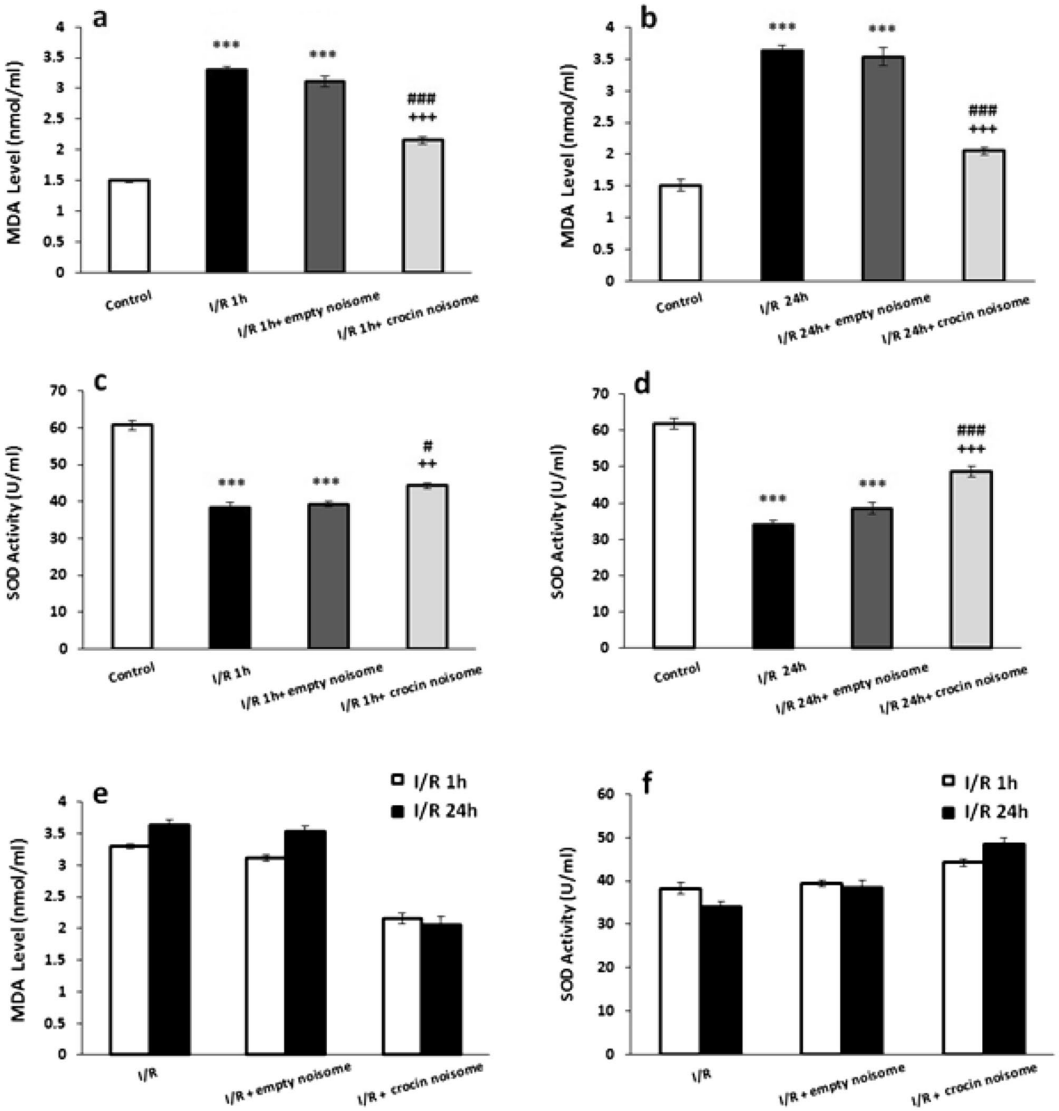
The effect of crocin-loaded nanoniosomes on (**a**) 1 h after I/R-induced changes in MDA serum level, (**b**) 24 h after I/R-induced changes in MDA serum level, (**c**) 1 h after I/R-induced changes in SOD serum level, (**d**) 24 h after I/R-induced changes in SOD serum level (**e**) comparison ischemia-induced changes followed by 1 h and 24 of reperfusion in MDA serum level and (**f**) comparison ischemia-induced changes followed by 1 h and 24 of reperfusion in SOD serum level. Data presented as mean ± SEM. ***P < 0.001 versus control group; ^++^P < 0.01 and ^+++^P < 0.001 versus I/R group; ^#^P < 0.05 and ^###^P < 0.001 versus I/R + empty niosome group.

Based on Fig. 5, renal ischemia followed by 1 h or 24 h of reperfusion significantly decreased SOD activity as compared with the control group (P < 0.001). Reduction of SOD activity in ischemic rats followed by 1 h (P < 0.01, Fig. 5a) or 24 h (P < 0.001, Fig. 5b) of reperfusion was significantly improved by crocin-contain nano-niosome treatment. However, MDA level significantly increased after ischemia followed by 1 h or 24 h of reperfusion as compared to the control animals (P < 0.001). The administration of crocin-loaded nano-niosomes suppressed the increased level of MDA in rat’s serum compared with the untreated animals (P < 0.001) (Fig. 5c,d). There was no significant difference between I/R group (P = 0.585), I/R + empty niosome (P = 0.097) and I/R + crocin noisome (P = 0.261) groups in SOD activity after ischemia followed by 1 h and 24 h of reperfusion (Fig. 5e). In addition, no significant difference was observed between I/R group (P = 0.522), I/R + empty niosome (P = 0.153) and I/R + crocin noisome (P = 0.895) groups in MDA level after ischemia followed by 1 h and 24 h of reperfusion (Fig. 5f).

### The effect of crocin-loaded nano-niosomes on I/R-induced histological damages

**Figure 6:**
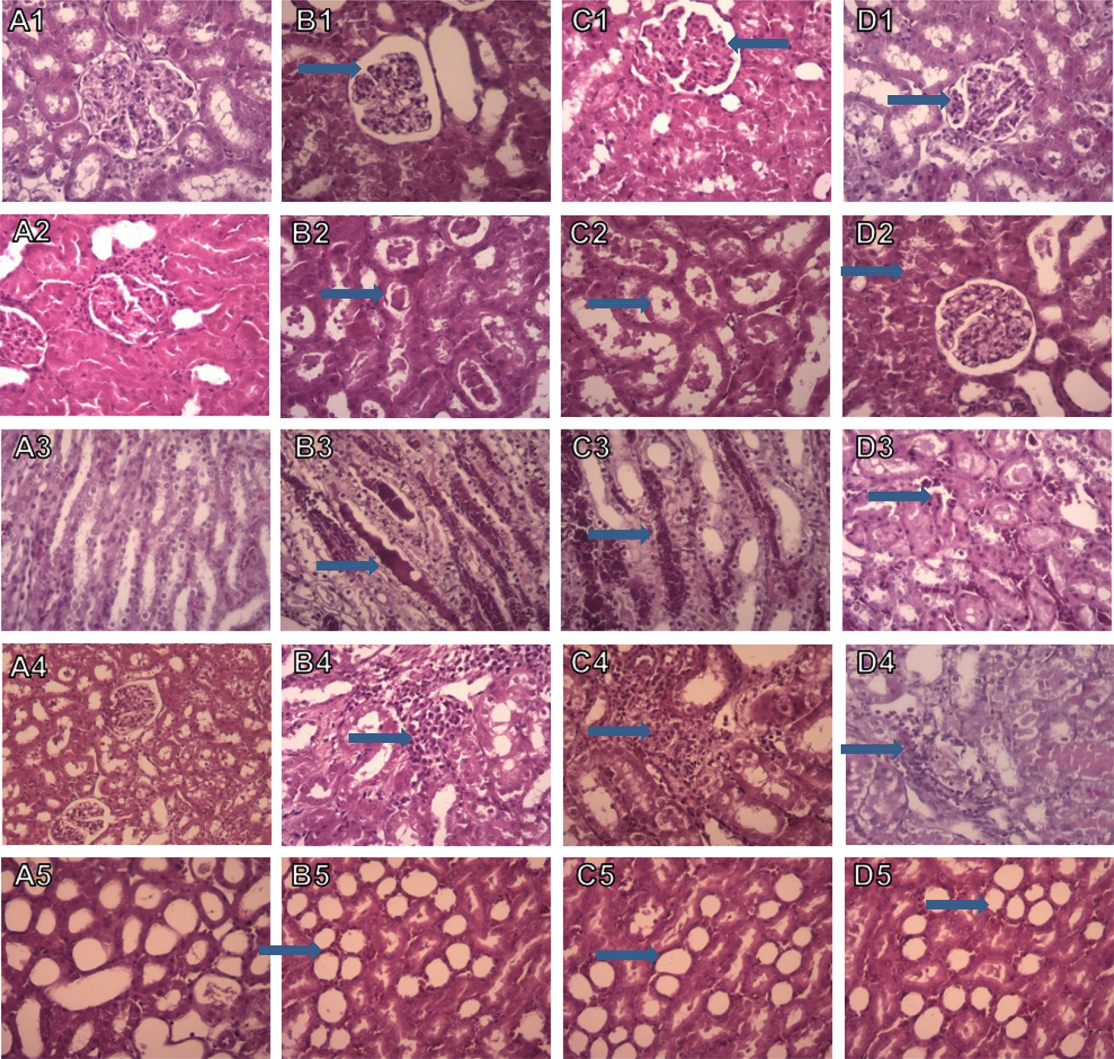
Light photomicrographs of the rats kidney sections (H&E). Kidney sections from control group showed normal renal morphology (**A1**–**A5**). Histological damages after ischemia followed by 1 h of reperfusion including reduction in the size of glomerular tuft, necrosis, medullary vascular congestion and inflammation and cast were observed in I/R (**B1**–**B5**) and I/R empty niosome (**C1**–**C5**) groups. The administration of crocin-loaded nanoniosomes attenuated these damages compared with untreated animals (Magnification ×400).

**Figure 7:**
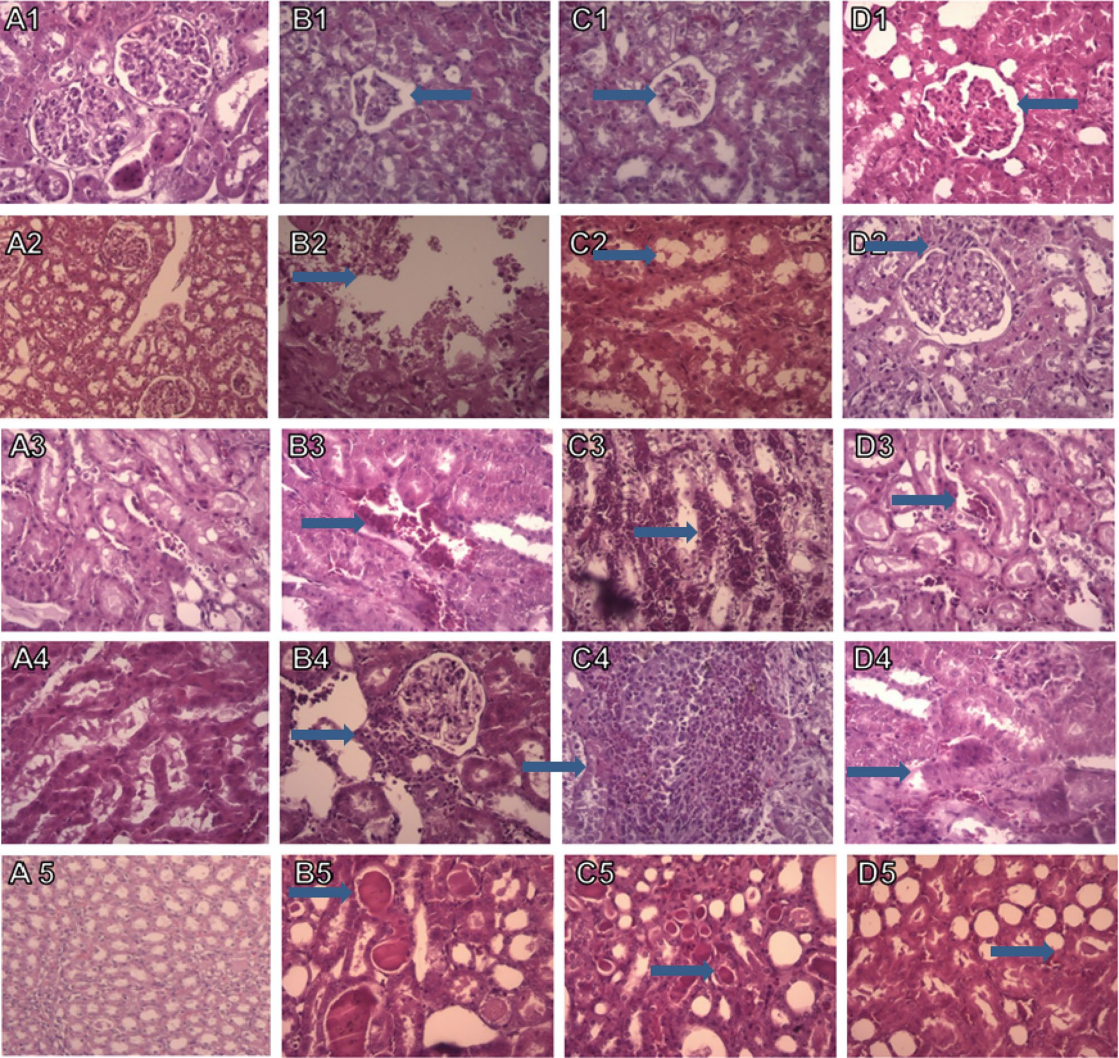
Light photomicrographs of the rats kidney sections (H&E). Kidney sections from control group showed normal renal morphology (**A1**–**A5**). Renal ischemia followed by 24 h of reperfusion caused severe histologic damages including reduction in the size of glomerular tuft, necrosis, medullary vascular congestion, inflammation and cast were observed in I/R (**B1**–**B5**) and I/R empty niosome (**C1**–**C5**) groups. These I/R-induced histologic damages were reduced in animals treated with crocin-contain nanoniosomes compared with untreated groups (Magnification ×400).

Renal tissue sections had a normal morphology in the control group. The renal tissue of rats subjected to ischemia followed by 1 h of reperfusion showed the recognized features of ischemic injury, including the reduced size of glomerular tuft, necrosis and exfoliation of the proximal tubular cells into the lumen, dilatation and congestion of blood vessels, medullary vascular congestion, tubular cast, and inflammation. These I/R-induced histologic damages were mitigated in animals treated with the niosomal formulation containing crocin compared to the untreated groups. Renal ischemia followed by 24 h of reperfusion caused more severe histologic damages, and treatment with crocin-containing niosomal formulation relieved the I/R-induced damages in the kidney. The histopathological alterations induced by ischemia followed by 1 h and 24 h of reperfusion are graded and summarized in Table 1, respectively. Light microscopic evaluations of the renal tissue sections post-ischemia followed by 1 h and 24 h of reperfusion are presented in Figs. 6 and 7, respectively.

**Table 1:** The effect of niosomal formulation contain crocin on I/R-induced morphological changes followed by 1 h and 24 h of reperfusion.

Groups	Reduction size of	Necrosis	Medullary vascular cogestion	Inflammation	Tubular cast
Control 1 h	0.4	0	0.6	0	0
I/R 1 h	1	0.8*	1.4	1	0
I/R empty noisome	0.8	0.8*	1.8	0.8*	0
I/R crocin noisome	0.4	0.4	1.8	1	0
Control 24 h	0	0	1	0	0
I/R 24 h	1.5**	1	1.8	1.5**	2**
I/R empty 24 h noisome	1.4**	0.6	2.2**	1.6**	2**
I/R crocin 24 h noisome	1	0.5	1**	0.5^**+**^	1.5

*P < 0.05, **P < 0.01 versus control; ^+^P < 0.05 versus I/R and I/R + empty niosome group.

The present study was conducted to formulate crocin-containing nano-niosomes and evaluate their effects on the serum levels of creatinine and urea in an in vivo rat model of renal I/R. In addition, the effect of crocin nano-niosomes was investigated on serum MDA and SOD activity and the possible hepatorenal protective effects. The investigation of physicochemical properties of crocin-contain nano-niosomes showed that they have appropriate size and morphology. Furthermore, nano-niosomes demonstrated acceptable encapsulation efficiency and release pattern of crocin. The in vitro results indicated that renal ischemia followed by 1 h or 24 h of reperfusion increased creatinine, urea, and MDA serum levels and decreased SOD activity. In addition, ischemia–reperfusion caused extensive damage to the kidney tissue. Treatment with crocin-loaded nano-niosomes reduced creatinine, urea, and MDA serum levels, while these nano-carriers raised SOD activity in the ischemic rat model. Furthermore, I/R-tissue damages were mitigated following the administration of crocin-containing nano-niosomes.

Recently, nano-carriers have gained attention as useful drug delivery systems for disease treatment. Niosome nano-carriers are non-ionic surfactants with numerous applications in drug delivery. Niosomes exhibit nontoxicity and non-immunogenicity, and these vesicles can be easily prepared at a low cost. In addition, they enhance drug potency and have greater chemical stability than other lipid vesicles^7,8^. Niosomes also act as a source for releasing drugs in a steady, controlled, and sustained delivery approach. These useful characteristics of niosomes make them excellent candidates for delivery applications. In addition, it has been demonstrated that crocin-coated magnetite nanoparticles are able to upregulate of apoptotic cells and downregulate of Bcl-2 labeling and markers of cell proliferation, inflammation, oxidative stress and angiogenesis^28^. Ischemia/reperfusion in the kidney is characterized as a pathophysiological condition occurring due to temporary restriction of blood to the kidneys and followed by increased production of free radicals and oxidative stress. Therefore, antioxidant compounds are believed to improve I/R-induced damages.

Pharmacological studies have demonstrated that saffron and its components, including crocin, possess anti-microbial, anti-depressant, anti-inflammatory, anti-tumor, chemopreventive properties^12,13,16^. Crocin has free radical scavenging and antioxidant activities and can promote the diffusion of oxygen in different tissues^10,11^. Saffron is shown to exert positive effects on kidneys; this plant acts as a diuretic and purifies the kidneys and bladder. It can also mitigate urinary tract infection and facilitate the passage of renal stones^26^. Assimopoulou et al. reported that crocin has high radical scavenging activity^10^. Ashktorab and colleagues have reported saffron can relieve various disorders through its anti-oxidant and anti-inflammatory effects, inhibition of cell proliferation, induction of apoptosis, and genoprotective properties^29^. Numerous studies have demonstrated that this potent antioxidant exerts protective effects in ischemia–reperfusion models of the brain, skeletal muscles, heart, retina, gastric mucosa, and kidneys in rats^18,20–22,27,28^. Hosseinzadeh et al. concluded that antioxidant capacity decreased in kidneys exposed to ischemia (60 min) followed by reperfusion (90 min). The administration of aqueous saffron extract and crocin increased the levels of lipid peroxidation products and antioxidant power in rat kidneys^22^. Previous studies have demonstrated that crocin can alleviate diabetes-induced renal injuries. Pretreatment with crocin decreased the high serum level of Cr and BUN, MDA production, and XO activity, while it increased GSH levels in diabetic rats^29,30^. It has been reported that 24 h of reperfusion following 30 min of renal ischemia increased the plasma levels of creatinine and urea nitrogen. Crocin reversed creatinine and urea-nitrogen levels by increasing renal blood flow, reducing cell injuries, and decreasing the pressure in Bowman's space. It also decreased lipid peroxidation and enhanced antioxidant capacity in the kidney tissue of rats^31^. Mard et al. reported that crocin protects the kidney tissue against I/R injury by decreasing the serum levels of AST, ALT, and BUN, increasing antioxidant enzyme activities of superoxide dismutase (SOD), catalase (CAT), and glutathione peroxidase (GPX) enzymes, and reducing hemorrhage and congestion of kidney blood vessels^32^. Treatment with crocin decreased the plasma level of creatinine, urea, and MDA in rats with acute renal failure. It also increased total antioxidant capacity and GSH peroxidase concentration in kidney tissue^24^. Pretreatment with saffron extract had protective effects against ischemia/reperfusion-induced acute kidney injury (AKI) by decreasing plasma creatinine concentration and malondialdehyde level^33^. The ischemia-induced histological damages in the kidneys include a reduced size of glomerular tuft, enlarged Bowman’s space, loss of brush borders, congestion and dilatation of blood vessels, necrosis, and exfoliation of the proximal tubular cells into the lumen^34–36^. Previous studies have shown that antioxidant agents ameliorate histopathological lesions caused by ischemia in rat kidney tissues^37–39^. Mard et al. reported that pretreatment with crocin reduced the histopathological disturbance of renal tissue, including hemorrhage and congestion of blood vessels, following IR-induced hepatic injury^32^. In addition, based on histopathological studies, crocin prevented proximal tubules' damage and necrosis in cisplatin-induced acute renal failure^24^.

## Conclusion

Our findings revealed that crocin-loaded nano-niosomes formulated via the modified heating method mitigated I/R-induced damages in an in vivo rat model of renal ischemia. These nano-carriers improved renal function, attenuated oxidative damages, and reduced histopathological changes in ischemic rats. Crocin-loaded nano-niosomes attenuated the observed effects of I/R in the kidney. Overall, the results indicated that crocin-loaded nano-niosomes have improving effects on renal I/R-induced injuries which are exerted, at least in part, through the antioxidant activities of crocin nano-carriers. The nano-niosomes were characterized by SEM, TEM, DLS, and FT-IR.
